# Genome-Wide Identification and Characterization of the *MAG2* Gene Family in *Medicago sativa*

**DOI:** 10.3390/genes17091111

**Published:** 2026-09-12

**Authors:** Caiqin Xu, Tao Zhou, Ying Huang, Yihan Yang, Jing Liu, Lu Yang, Qian Li, Xiqiang Liu, Bo Zhang

**Affiliations:** 1 Key Laboratory of Ministry of Education of Grassland Resources and Ecology of Western Arid Region, Key Laboratory of Grassland Resources and Ecology of Xinjiang, College of Grassland Science, Xinjiang Agricultural University, Urumqi 830052, China; niq0613@163.com (C.X.);; 2 Key Laboratory of State Forestry and Grassland Administration on Desert Oasis Ecosystem Protection and Restoration, Xinjiang Key Laboratory of Fruit Tree Species Breeding and Cultivation, Xinjiang Academy of Forestry, Urumqi 830000, China; 3 Institute of Ecological Protection and Restoration, Chinese Academy of Forestry/Grassland Research Center, National Forestry and Grassland Administration, Beijing 100091, China

**Keywords:** *MAG2* gene family, genome-wide identification, vesicular transport, *Medicago sativa*

## Abstract

**Background/Objectives:** Vesicular trafficking mediates the transport of proteins and other cellular components between intracellular organelles. The MAG2 complex serves as a key tethering factor mediating ER–Golgi retrograde vesicle transport, and has been implicated in plant growth, development, and stress responses. **Methods:** In this study, seven *MAG2* family genes were identified in *Medicago sativa* from the Zhongmu No. 4 reference genome using hidden Markov model searches (HMM) and BLASTP analysis. **Results:** Phylogenetic analysis assigned four genes to the MAG2 subfamily and three genes to the MAG2L subfamily. Chromosomal distribution and collinearity analyses indicated that segmental duplication potentially contributed to the expansion of the MAG2 family in alfalfa. Gene structure and conserved motif analyses revealed a high degree of conservation among the identified members, with Motif 10 occurring specifically in the MAG2 subfamily. Subcellular localization prediction analysis predicted that four MsMAG2 proteins were localized in chloroplasts, two in the nucleus, and one in the peroxisome. Analysis of promoter sequences identified numerous cis-regulatory elements associated with responses to hormones, light, and environmental stress, suggesting that *MsMAG2* genes may participate in plant development and environmental adaptation. Furthermore, genome-wide association analysis identified *MsMAG2L2* that can be associated with tillering. **Conclusions:** These results provide a comprehensive genome-wide characterization of the *MAG2* gene family in alfalfa and establish a basis for further investigating the functional associations of MsMAG2L2 in branch development.

## 1. Introduction

Eukaryotic cells contain numerous organelles enveloped by phospholipid bilayers, which enable specific biochemical reactions to occur accurately and efficiently within relatively independent spaces [1,2]. The exchange of proteins, lipids, and other cellular components between membrane-bound organelles depends largely on vesicular transport, which maintains organelle identity and coordinates intracellular material distribution [3,4,5]. This transport process involves the directional movement of various membrane vesicles along with their complex regulatory mechanisms. The specificity of vesicle transport relies on the functions of multiple conserved transmembrane and soluble proteins. The vesicle transport process primarily includes: the budding of transport vesicles from donor membranes; the directional movement of transport vesicles along the cytoskeleton or via diffusion; the tethering of transport vesicles to target membranes; and the anchoring and fusion of the target membrane with the transport vesicle membrane [6,7]. Each step in the process is mediated by one or several specific proteins. Among these, the recognition and fusion between transport vesicles and target membranes require several conserved regulatory factors, primarily including tethering factors, ARF (ADP-Ribosylation factors), SNARE (soluble N-ethyl maleimide-sensitive factor attachment protein receptors), and GEF (guanine nucleotide exchange factors), which collectively regulate the vesicle transport process [8,9,10,11,12]. In plants, vesicular transport is further coordinated by conserved Rab and ARF GTPase regulatory systems within the endomembrane network [13,14]. The tethering factor facilitates the initial contact between vesicles and target membranes during vesicle transport, a process supported by the tethering protein complex, which occurs in membrane systems such as the Golgi apparatus, the ER–Golgi interface, and vacuoles. Tethering factors are crucial for vesicle transport and are also vital for the growth and development of plants. These factors can be divided into two major categories: one consists of long coiled-coil protein molecules, including GM130 and p115 [7,15], while the other comprises multisubunit tethering complexes (MTCs), such as the *Arabidopsis* MAG2 complex, the COG (conserved oligomeric Golgi) complex, the GARP (Golgi-associated retrograde protein) complex, the TRAPP (transport protein particle) complex, the HOPS (homotypic fusion and vacuole protein sorting) complex, and the exocyst complex [16,17,18,19,20].

Different tethering factors are localized to distinct organelles and function as specific recognition mechanisms. For instance, the Exocyst complex is an evolutionarily conserved octameric protein complex composed of eight subunits: Sec3, Sec5, Sec6, Sec8, Sec10, Sec15, Exo70, and Exo84. This complex mediates the tethering process of post-Golgi vesicles to the plasma membrane [21]. Although there is a preliminary understanding of tethering factors and their functions in yeast and mammals, research on plant tethering factors remains in its infancy. During the screening process for *Arabidopsis* mutants exhibiting abnormal storage protein transport, the endoplasmic reticulum tethering factor MAG2 was identified. Loss-of-function mutations in the *MAG2* gene result in blocked vesicle transport between the endoplasmic reticulum and the Golgi apparatus, leading to the accumulation of storage protein precursors within the endoplasmic reticulum and the formation of a novel cellular structure known as the MAG2 body [17]. Concurrently, due to the inability of a substantial amount of storage protein precursors to reach the vacuole for processing by vacuolar enzymes, a significant quantity of these precursors remain in mature seeds [17]. The MAG2 complex is an endoplasmic reticulum-localized retention complex that regulates vesicle transport between the Golgi apparatus and the endoplasmic reticulum. *MAL* (*MAG2*-*Like*) is a *MAG2* homolog in *Arabidopsis*. The seedlings of the *Arabidopsis mag2-1*×*mal*-1 double mutant exhibited shorter root lengths, reduced plant heights, smaller rosette leaves, seeds, and thousand-seed weight compared to the wild type, while the seedlings of *MAG2*-overexpressing plants demonstrated longer root lengths and greater plant stature. This indicates that MAG2 and MAL play crucial regulatory roles in plant growth and development. Analysis using DR5:GUS, PIN1:GUS, PIN2:GUS, and PIN3:GUS reporter lines revealed significant alterations in auxin distribution and auxin transporter localization in the *mag2-1* and *mal-1* single mutants, as well as in the *mag2-1*×*mal-1* double mutant. The results from the exogenous application of NAA and NPA indicated that both single and double mutants, along with overexpression lines, exhibited reduced sensitivity to 50 nM NAA treatment compared to the wild type. Additionally, the double mutant *mag2-1*×*mal-1* and overexpression lines displayed reduced sensitivity to 0.3 μM NPA treatment relative to the wild type. NPA is a well-characterized auxin transport inhibitor that blocks PIN protein cycling and vesicle trafficking [22,23], and the observed reduced sensitivity further implies that MAG2 and MAL modulate polar auxin transport through vesicle-mediated mechanisms. These findings suggest that MAG2 and MAL may regulate auxin homeostasis by controlling vesicle trafficking, thereby modulating plant development [24]. Furthermore, MAG2-interacting proteins MIP1, MIP2, and MIP3 were identified; these four protein molecules form a stable complex that collectively regulates retrograde transport between the endoplasmic reticulum and Golgi (ER–Golgi). The deletion mutants of MIP1, MIP2, and MIP3 exhibit phenotypes similar to those of the MAG2 deletion mutant, including the appearance of MAG2 bodies and the abnormal accumulation of storage protein precursors (12S globulin and 2S globulin) in mature seeds [25]. The growth and development of the *mag2-1*×*mip3-1* double mutant were significantly impaired, and it exhibited sensitivity to salt stress [26]. The MAG2 complex interacts with Qa-AtSYP81 and Qa-AtSec20, which are members of the endoplasmic reticulum-localized SNARE complex, and may collaboratively regulate vesicular transport from the Golgi apparatus to the endoplasmic reticulum. In addition to their crucial regulatory role in vesicle trafficking, MAG2 and MIP proteins are also implicated in plant responses to abiotic stresses, including salt, heat, and osmotic stress, and to phytohormones, including abscisic acid (ABA) and gibberellins [27,28]. The absence of one or both of MAL and MAG2 significantly impacts the stability of the MAG2 complex, indicating that MAL may interact with MIP proteins at different developmental stages or in various tissues, particularly when MAG2 is deficient, to modulate vesicle trafficking. This hypothesis necessitates further evidence for validation.

*Medicago sativa*, a perennial leguminous herb, is often referred to as the “King of Forages” due to its high protein and nutrient content [29]. It has been extensively introduced and cultivated worldwide. This study employed the Zhongmu No. 4 genome reference data to identify the *MAG2* gene family in alfalfa, and characterized its physicochemical properties, evolutionary relationships, chromosomal localization, and promoter regions [30]. Furthermore, the expression patterns of *MsMAG2* genes across various tissues were examined. This research lays a foundation for more detailed investigations into the functions of the *MsMAG2* gene in alfalfa and provides valuable genetic resources for molecular breeding in this species.

## 2. Materials and Methods

### 2.1. Identification and Sequence Analysis of the MAG2 Gene

The hidden Markov model of the MAG2 conserved domain (PF04437) was obtained from the PFAM database (https://www.ebi.ac.uk/interpro/entry/pfam/#table, accessed 8 June 2026). The genome data of *M. sativa* was downloaded from the online platform FigShare (https://figshare.com/articles/dataset/Medicago_sativa_genome_and_annotation_files/12623960, accessed 8 June 2026). Using HMM Search in TBtools v2.085 with a strict E-value threshold set at 1 × 10^−10^, genes containing the MAG2 conserved domain were identified from the alfalfa genome. Subsequently, the BLASTP tool was employed with an E-value threshold of 1 × 10^−10^ to align the protein sequences of alfalfa against the sequences of the *Arabidopsis MAG2* gene family. The results from HMM and BLASTP were merged and deduplicated to obtain the sequences of the alfalfa *MAG2* gene family. Candidate sequences were manually filtered to retain those with >50% coverage of the PF04437 domain and complete open reading frames (lacking truncations and containing canonical start/stop codons). The conserved MAG2 domain in the final seven proteins was further confirmed using the NCBI-CDD and SMART databases. A phylogenetic tree was constructed with the *MAG2* gene families of both alfalfa and *Arabidopsis*, designating genes that clustered with the *Arabidopsis MAG2* gene family as the alfalfa *MAG2* gene family. The protein sequences of the *Arabidopsis MAG2* gene family were sourced from Plant TFDB (https://planttfdb.gao-lab.org/, accessed 8 June 2026). The Expasy online tool (https://www.expasy.org/, accessed 8 June 2026) was utilized to analyze the isoelectric point (pI) and molecular weight (MW) of the *Medicago truncatula* MAG2 protein sequence. Finally, the protein sequence encoded by the *M. sativa MAG2* gene was submitted to the WoLF PSORT website (WoLF PSORT: Protein Subcellular Localization Prediction, accessed 8 June 2026) for predicting subcellular localization.

### 2.2. Phylogenetic Analysis and Subfamily Classification of MAG2 Genes in M. sativa and Arabidopsis

The protein sequences of *M. sativa* and *Arabidopsis* were analyzed using MEGA7.0 for multiple sequence alignment and trimming. The PHYLIP3.0 software package was selected to analyze the similarity of protein sequences, generating output files in PHYLIP format. The RAxML-HPC2 on XSEDE model was chosen on the CIPRES online platform (https://www.phylo.org/, accessed 8 June 2026), employing the Maximum Likelihood (ML) method with the operational model set to DAYHOFF and a bootstrap value of 1000 to execute the program. Visualization and enhancement of the phylogenetic tree were performed using iTOL (https://itol.embl.de/, accessed 8 June 2026) and Adobe Illustrator (https://www.adobe.com/products/illustrator.html, accessed 8 June 2026). The classification of the *Arabidopsis* MAG2 subfamily was referenced, and genes clustering with the *Arabidopsis* MAG2 subfamily were designated as members of that subfamily.

### 2.3. Motif and Structural Analysis of MAG2 Genes

The *MAG2* gene sequence of alfalfa was uploaded to the MEME website (https://meme-suite.org/meme/tools/meme, accessed 8 June 2026), setting the prediction to a maximum of 8 motifs. The GFF file containing the chromosome location information of alfalfa was input into TBtools (v1.098) to determine the UTR and CDS of each *MAG2* gene. Finally, TBtools was used to merge the results and conduct a visual analysis.

### 2.4. Chromosome Localization and Gene Collinearity Analysis

The alfalfa location information file was uploaded to TBtools and chromosome localization analysis was performed using the default parameters. This maps the alfalfa *MAG2* gene to 7 out of 32 chromosomes. Next, the alfalfa genome file and gene location information file were uploaded to the MCscanX software (v1.0) within TBtools. After executing the program, the gene density file, synteny file, and other relevant files were obtained. Finally, these files were uploaded to Advanced Circos (v1.1.2) to highlight the synteny of the *MAG2* gene.

### 2.5. Genome-Wide Association Study

This dataset utilized an association panel composed of 100 alfalfa germplasms to analyze the relationship between SNP markers and important phenotypic traits. In the variant detection analysis, a total of 65,773,899 SNPs were initially filtered through stringent criteria. Subsequently, VCFtools [31] was employed to screen for SNP loci with a genotype call rate ≥ 90%, minor allele frequency (MAF) ≥ 0.05, and biallelic nature, resulting in 5,653,034 high-quality SNPs. We conducted a genome-wide association analysis using the R package rMVP v1.4.6, incorporating two models: the general linear model (GLM) and the mixed linear model (MLM). Principal components were included as covariates in the model for adjustment. The threshold for significant loci (−Log_10_(P), logarithm of odds, LOD) was set at 5. The R package “CMplot” v4.5.1 was used for the visualization of GWAS results. Additionally, colocalization SNP quantity analysis and haplotype analysis were conducted for all significant SNP loci with LOD ≥ 5. For each SNP, the allele exhibiting superior phenotypic performance was selected as the superior allele.

### 2.6. Protein–Protein Interaction Network Analysis

A protein–protein interaction (PPI) network of MsMAG2L2 was predicted using the STRING database (https://cn.string-db.org/, accessed 8 June 2026), with the closely related species Medicago truncatula selected as the reference organism. The homologous protein A0A072V5P0 was used to represent MsMAG2L2 in the analysis. The minimum required interaction score was set to 0.4 (medium confidence), while all other parameters were maintained at their default settings. The resulting protein interaction network was visualized using STRING (v12.0).

## 3. Results

### 3.1. Identification of the MAG2 Gene Family

**Figure 1:**
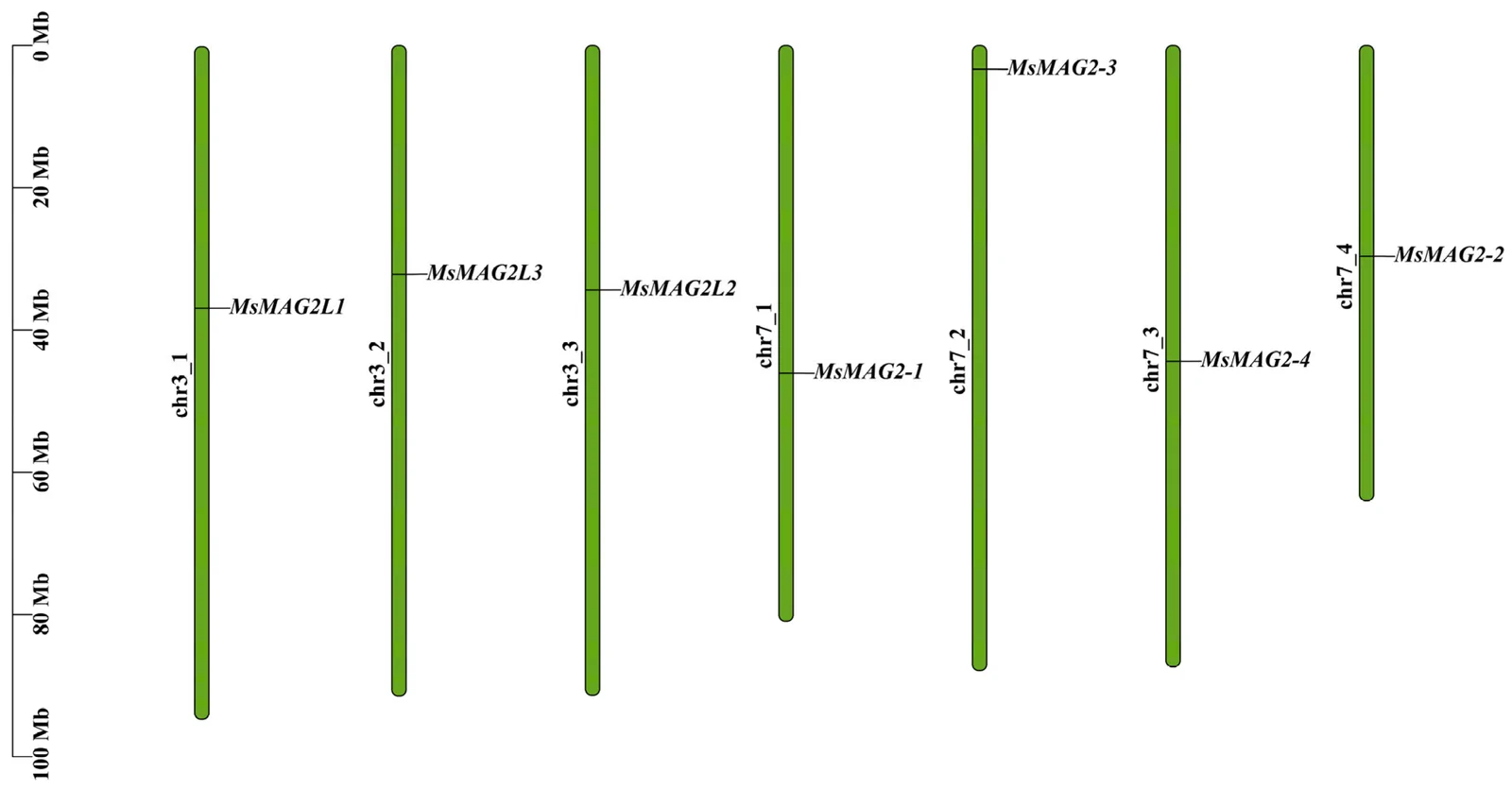
The chromosome distribution of *MAG2* genes in *M. sativa*. Chromosome names are labeled above each corresponding bar, while the vertical scale on the left indicates chromosome sizes in million base pairs (Mb).

This study identified a total of seven *MAG2* genes in alfalfa, designated as MsMAG2-1, MsMAG2-2, MsMAG2-3, MsMAG2-4, MsMAG2L1, MsMAG2L2, and MsMAG2L3. Subcellular localization prediction of these seven proteins revealed that two (MsMAG2L1 and MsMAG2L3) were predicted to be localized to the nucleus, four (MsMAG2-1, MsMAG2-2, MsMAG2-3, and MsMAG2-4) to the chloroplasts, and one (MsMAG2L2) to the peroxisome (Table 1). Among these seven MAG2 proteins, MsMAG2L2 comprises the shortest amino acid sequence, consisting of 505 amino acids, while MsMAG2L3 comprises the longest sequence with 839 amino acids. The predicted molecular weight ranges from 59,022.97 Da (MsMAG2L2) to 96,895.75 Da (MsMAG2L3). The isoelectric point prediction results indicate that the pI ranges from 5.52 (MsMAG2-2) to 6.66 (MsMAG2L1), with all proteins being acidic. Chromosomal mapping revealed that the seven *MAG2* genes were distributed across seven chromosomal haplotypes (Figure 1). The distribution of these genes across multiple haplotypes of the same chromosome (e.g., chr3_1, chr3_2, chr3_3) reflects the autotetraploid nature of the alfalfa genome, where homologous chromosomes are represented as distinct haplotypes. Among them, MsMAG2L1, MsMAG2L2, and MsMAG2L3 are located on chr3_1, chr3_3, and chr3_2 of chromosome 3, respectively, while MsMAG2-1, MsMAG2-2, MsMAG2-3, and MsMAG2-4 are located on chr7_1, chr7_4, chr7_2, and chr7_3 of chromosome 7, respectively. The *MAG2* genes are distributed exclusively on chromosomes 3 and 7, with no distribution observed on other chromosomes.

### 3.2. Phylogenetic Structure of MAG2 Proteins

**Figure 2:**
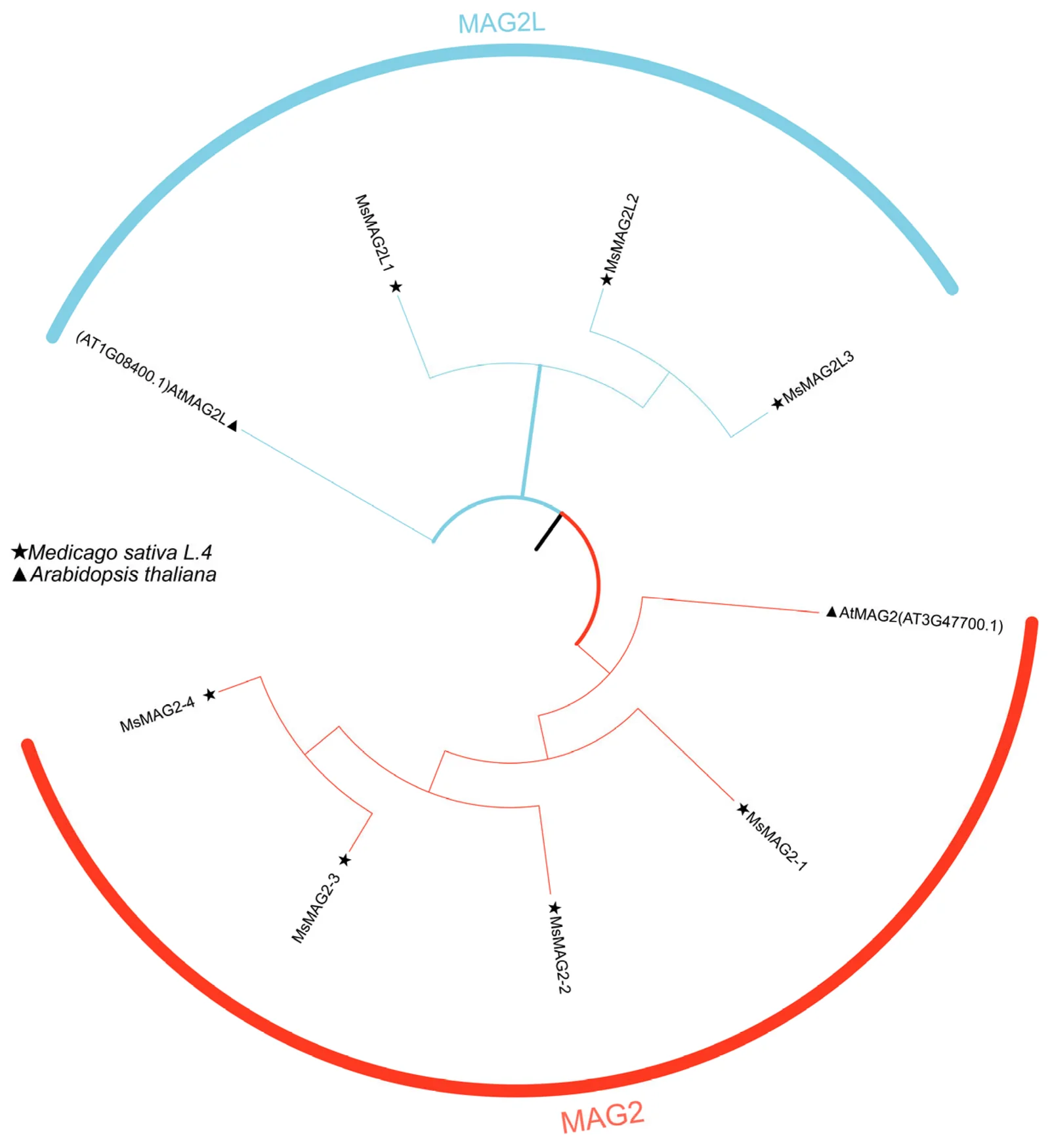
Phylogenetic evolutionary tree of *MAG2* genes in *M. sativa* and *Arabidopsis*. (★ represents *M. sativa*, ▲ represents *Arabidopsis*).

The phylogenetic tree of MAG2 proteins in *M. sativa* and *Arabidopsis* was constructed using MEGA7.0 (Figure 2). The results indicated that the seven MsMAG2 proteins were categorized into two major subfamilies: CLASS I and CLASS II. CLASS I is also referred to as the MAG2 subfamily, while CLASS II is known as the MAG2-like (MAG2L) subfamily. In contrast to *Arabidopsis*, which contains only one AtMAG2 and one AtMAG2L member, the MAG2 subfamily in *M. sativa* comprises four members, accounting for 57.1% of all MsMAG2 proteins. This is followed by the MAG2L subfamily, which includes three members, representing 42.9% of all MsMAG2 proteins. Notably, the four members of the MAG2 subfamily are exclusively found on chromosome 7 (chr7_1, chr7_4, chr7_2, and chr7_3), whereas the three members of the MAG2L subfamily are solely located on chromosome 3 (chr3_1, chr3_3, and chr3_2). Each haplotype contains only one member, suggesting that segmental duplication may be the primary mechanism driving the expansion of the *MAG2* gene family.

### 3.3. Conservative Motif and Gene Structure Analysis

**Figure 3:**
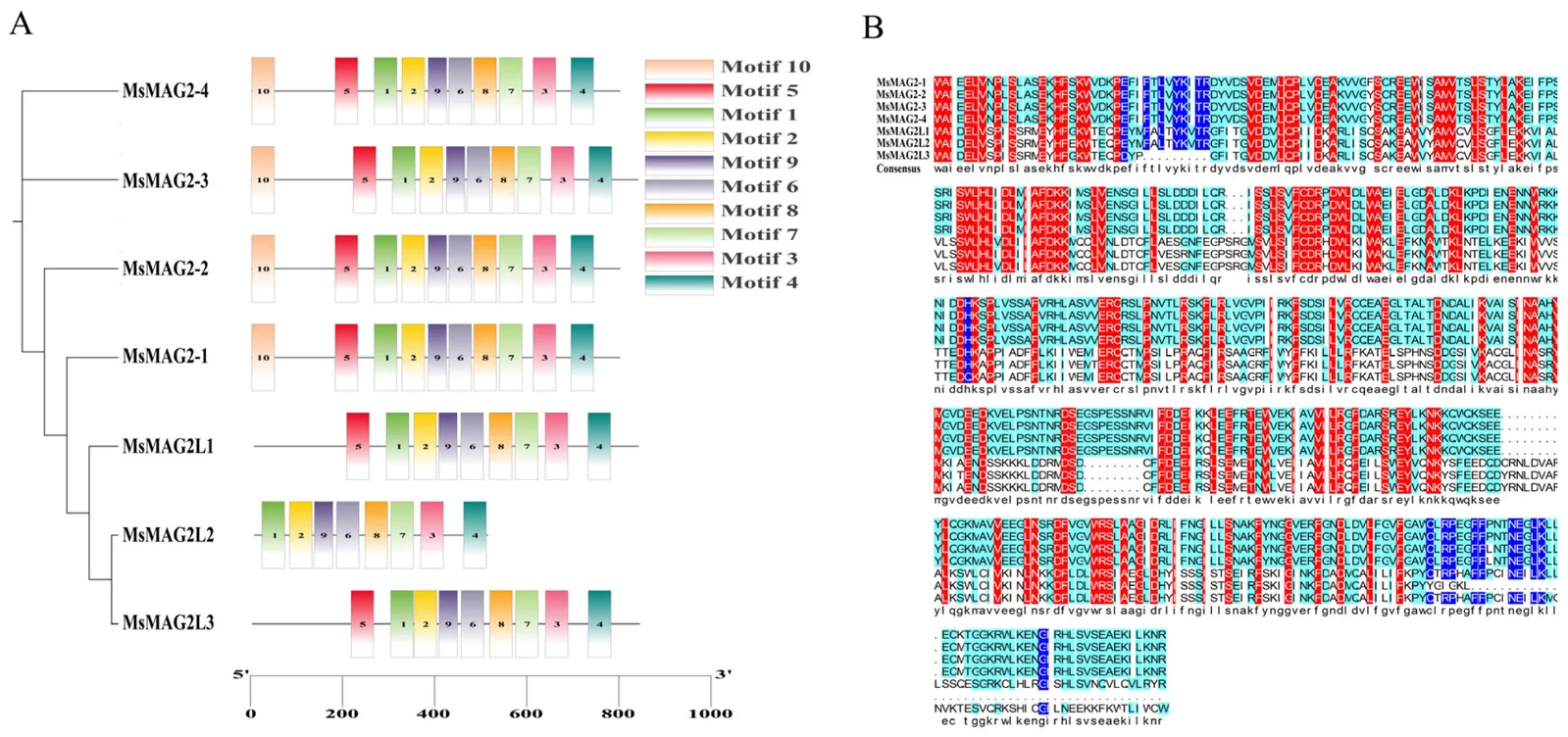
The conserved motif composition and gene structure of the *MsMAG2* gene system evolutionary relationship. (**A**) Motif distribution of MsMAG2 proteins, with different colors representing different motifs. (**B**) *MsMAG2* gene sequence alignment in *M. sativa*.

We predicted ten motifs in the protein sequences of MAG2 proteins and visualized these motifs using TBtools (Figure 3). The results indicated that all MAG2 proteins contain Motif1, Motif2, Motif3, Motif4, Motif6, Motif7, Motif8, and Motif9, suggesting that these motifs may play a critical role in the structural and functional characteristics of MAG2 proteins. All members of the MAG2 subfamily possess Motif10, whereas none of the members of the MAG2L subfamily do, implying that Motif10 is significant for the functional specificity of the MAG2 subfamily possess it. This subfamily-specific distribution suggests that Motif10 may be associated with the functional differentiation between the two subfamilies, although direct evidence for its functional role is not yet available. Additionally, Motif5 is not consistently present within the MAG2L subfamily, despite the presence of some cross-subfamily variation.

### 3.4. Gene Collinearity Analysis

**Figure 4:**
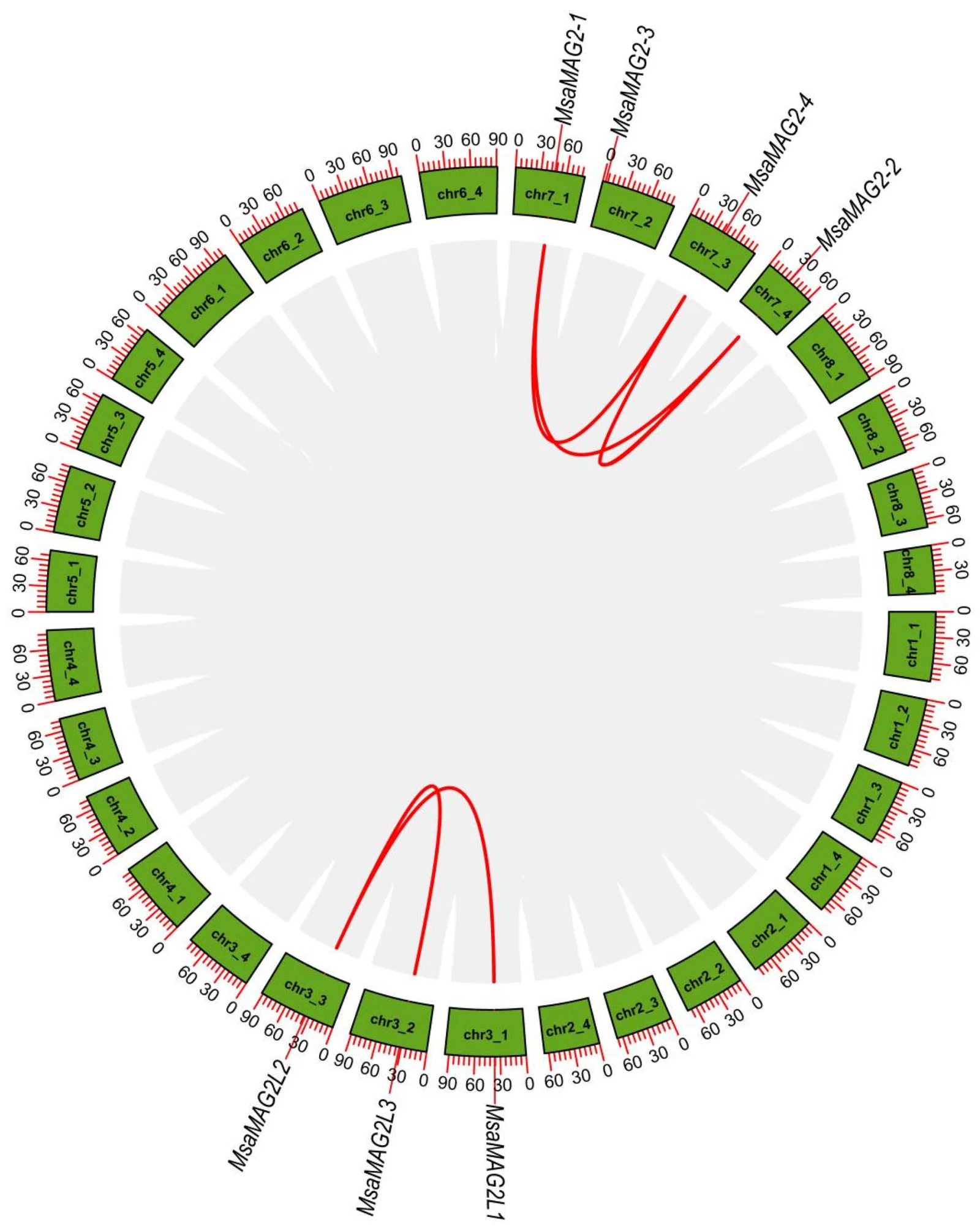
Genome-wide duplication and synteny analysis of *MsMAG2* genes. Homologous gene pairs derived from genome-wide duplication are connected by gray lines. Red lines specifically indicate segmental duplication events involving *MsMAG2* members.

Whole-genome duplication, gene tandem duplication, and segmental duplication are the three primary mechanisms responsible for the expansion of gene families. Synteny analysis within species is frequently employed to determine the presence of tandem or segmental duplication among genes. To gain a deeper understanding of the gene duplication events within the *MAG2* gene family, we performed a synteny analysis of the seven *MAG2* genes (Figure 4). The results of the collinearity analysis indicated that there are seven pairs of genes exhibiting collinear relationships: specifically, *MsMAG2-1* on chromosome chr7_1 and *MsMAG2-4* on chromosome chr7_3; *MsMAG2-1* on chromosome chr7_1 and *MsMAG2-2* on chromosome chr7_4; *MsMAG2-4* on chromosome chr7_3 and *MsMAG2-2* on chromosome chr7_3; *MsMAG2L2* on chromosome chr3_3 and *MsMAG2L3* on chromosome chr3_2; and *MsMAG2L2* on chromosome chr3_3 and *MsMAG2L1* on chromosome chr3_1. Collinearity analysis alone suggests that segmental duplication may have contributed to the expansion of the *MAG2* gene family in alfalfa, but confirmation through additional analyses such as Ks calculation is required. Notably, collinearity among *MAG2* genes was observed only within individual subfamilies, with no collinearity detected between genes of different subfamilies. Furthermore, collinearity was present among all genes within both the MAG2 and MAG2L subfamilies, indicating a high degree of conservation of the *MAG2* gene family throughout evolution, which aligns with the results of the Motif analysis. This may imply that the subfamilies of *MAG2* genes in alfalfa exhibit substantial conservation during the evolutionary process.

### 3.5. Analysis of Cis-Acting Elements

**Figure 5:**
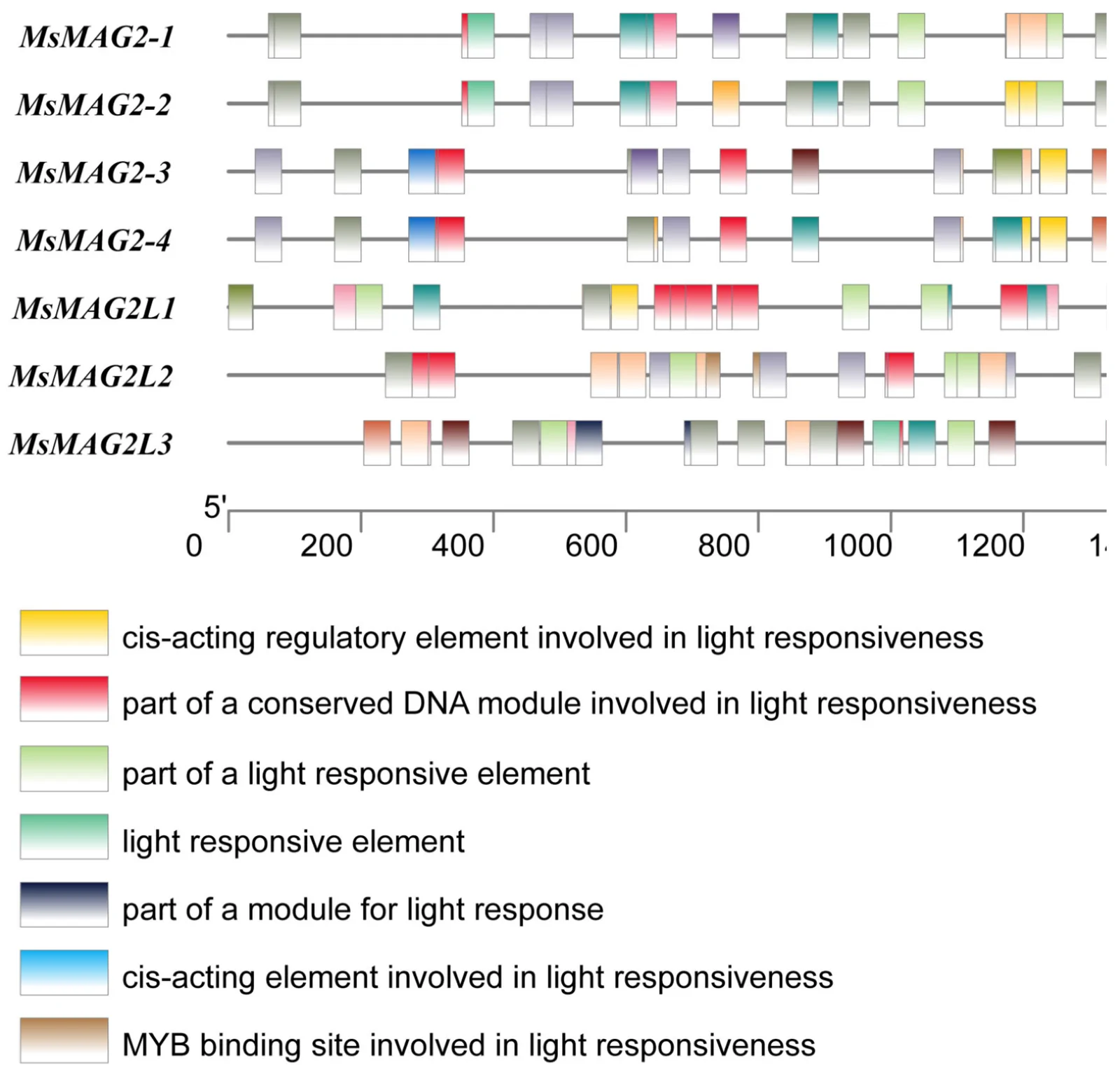
The cis-acting element analysis of 7 *MsMAG2* gene promoters. The *MsMAG2* genes were classified according to their phylogenetic relationships. The response elements are indicated by 22 differently colored columns, where each column represents a distinct element type. The horizontal coordinates correspond to gene length.

Through the analysis of cis-regulatory elements in the seven MsMAG2 promoters (2000 bp upstream of the transcription start site), we discovered that they contain several basic elements, such as the F-box element, along with a significant number of binding sites for MYC and MYB transcription factors. The analysis of elements associated with each gene revealed that the *MsMAG2-4* gene possesses the most functional elements (53), followed by the *MsMAG2-3* gene with 47 elements, the *MsMAG2-1* gene with 41 elements, the *MsMAG2L2* gene with 38 elements, and both the *MsMAG2L3* and *MsMAG2-2* genes with 33 elements each. The *MsMAG2L1* gene has the fewest elements (29). Additionally, a total of 144 elements related to light response, hormone response, and stress response were identified across the seven promoters (Figure 5). Among these, hormone response elements were the most abundant, totaling 86, followed by 61 light response elements, while stress response elements were the least numerous, with only 17. This suggests that *MsMAG2* genes may be regulated by plant hormones and light. Within the plant hormone response elements, jasmonic acid response elements were the most prevalent (45), followed by abscisic acid response elements (28), as well as salicylic acid response elements (7) and auxin response elements (5), with gibberellin response elements being the least abundant (1). This indicates that *MsMAG2* genes are primarily regulated by jasmonic acid and abscisic acid, with other hormones potentially playing a role in their regulation. Among the stress-related elements, there are 8 cold stress response elements and 6 drought response elements, suggesting that certain *MAG2* genes may be involved in the regulation of cold tolerance and drought resistance in plants. This distribution suggests that cis-regulatory elements associated with jasmonic acid and abscisic acid are preferentially enriched in the promoters of *MsMAG2* genes; however, such in silico predictions serve as a foundational genomic framework indicating potential regulatory capacities, whereas distinguishing between regulation by endogenous hormonal signals and responses to exogenous hormone applications requires direct physiological validation.

### 3.6. Genome-Wide Association Analysis

**Figure 6:**
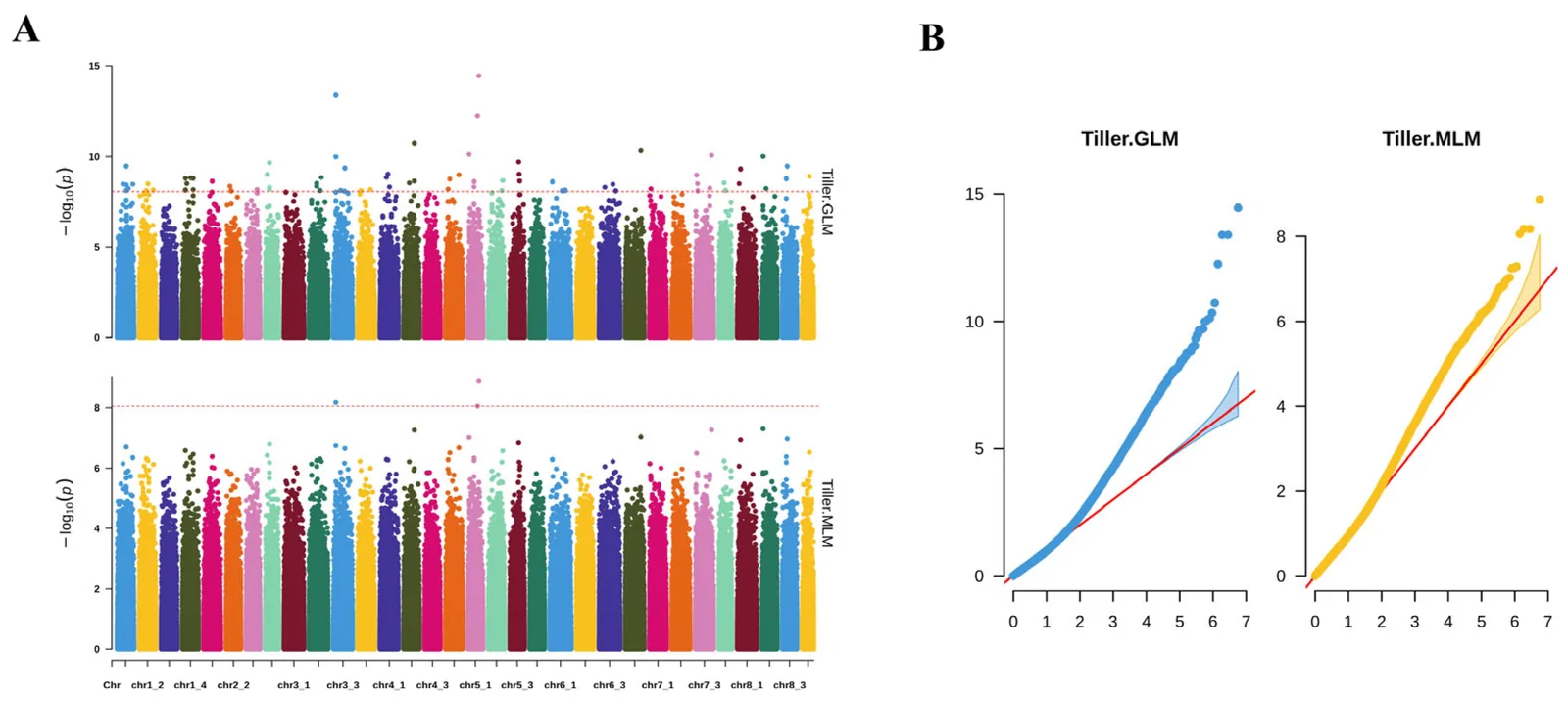
Manhattan Plot and Q-Q Plot for Association Analysis of Tillering Traits. (**A**) Manhattan plots for the GLM and MLM models. The x-axis represents chromosomal position, the y-axis represents significance, and the red dashed line indicates the threshold. (**B**) Q-Q plots for the GLM and MLM models. The red line represents the expected value.

Using previously generated data from the research group, a population of 100 alfalfa germplasms was utilized to analyze the relationship between SNP markers and tillering traits. The resequencing data were aligned to the ‘Zhongmu No. 4’ reference genome and filtered using VCFtools [31], resulting in the identification of 5,653,034 high-quality SNPs. Subsequently, a genome-wide association analysis was performed on the tillering traits. A significant SNP located at chromosome chr3_3:34372876 was identified (Figure 6), and through gene annotation and alignment analysis, it was determined to be located within the *MsMAG2L2* gene (*Msa0449710*). The GWAS result suggests that *MsMAG2L2* can be associated with tillering, although this association does not demonstrate a direct regulatory role.

### 3.7. Predicted Protein–Protein Interaction Network of MsMAG2L2

**Figure 7:**
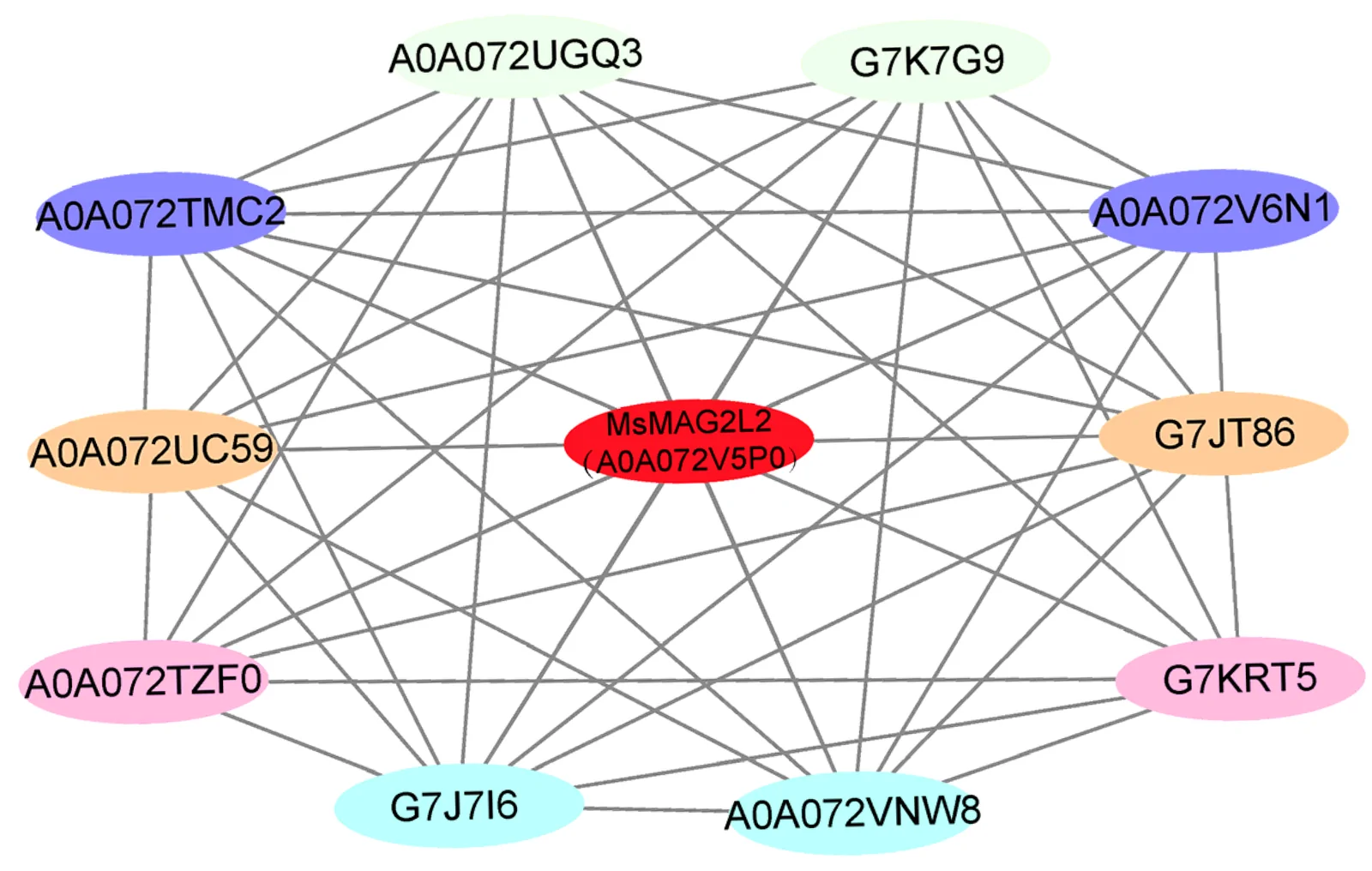
Predicted protein–protein interaction network of MsMAG2L2.

The predicted protein–protein interaction network indicated that the Medicago truncatula homolog of MsMAG2L2, A0A072V5P0, was associated with ten proteins (Figure 7), with significant PPI enrichment (*p*-value < 1.0 × 10^−16^). Among these predicted interaction partners, A0A072TMC2 was annotated as the membrane fusion protein Use1, whereas G7JT86 was annotated as a putative vesicle transport protein. The functional annotation of these interacting proteins suggests that MsMAG2L2 may be involved in membrane fusion and vesicle trafficking-related processes.

## 4. Discussion

Gene duplication is an important driving force in the expansion and diversification of plant gene families. In the present study, seven MAG2 family members were identified in alfalfa and classified into the MAG2 and MAG2L subfamilies based on their phylogenetic relationships with *Arabidopsis* homologs. Compared with *Arabidopsis*, which contains fewer MAG2-related members, alfalfa possesses a relatively expanded MAG2 family, which may be associated with its autotetraploid genome background and the retention of duplicated genes during genome evolution [32,33,34]. In addition, the restricted but uneven distribution of *MsMAG2* genes on chromosome 3 and chromosome 7-related haplotypes, together with the collinearity relationships observed within each subfamily, suggests that segmental duplication may have contributed to the expansion of this gene family in alfalfa. However, direct evidence from additional analyses, such as Ks calculation and comparison with other legume genomes, would be required to fully confirm this conclusion. Despite variation in copy number, MsMAG2 family members displayed relatively conserved gene structures and motif compositions. Closely related genes shared similar exon–intron organizations and conserved motifs, indicating that these genes have remained structurally stable during evolution. Notably, Motif 10 was present only in members of the MAG2 subfamily, suggesting that this motif may be associated with subfamily differentiation, though its functional significance requires experimental validation. Previous studies have demonstrated that MAG2 and MAL function as components of a tethering complex involved in vesicle trafficking between the Golgi apparatus and the endoplasmic reticulum [17,25], and that other tethering-related factors such as MAG4/Atp115 also participate in Golgi-associated transport in *Arabidopsis* [35]. In addition, efficient trafficking between the endoplasmic reticulum and the Golgi apparatus is central to membrane transport control in eukaryotic cells [36]. Therefore, the structural conservation observed in the MsMAG2 family may reflect the functional conservation of MAG2-related proteins in vesicle transport processes. This interpretation is also consistent with the general conservation of plant endomembrane trafficking pathways, including endosomal trafficking and trans-Golgi network-associated transport [37,38,39].

It is noteworthy that the predicted subcellular localizations of the seven MsMAG2 proteins—chloroplasts, nucleus, and peroxisome—differ from the ER–Golgi localization reported for the *Arabidopsis* MAG2 complex. This discrepancy most likely reflects the inherent limitations of sequence-based prediction algorithms, which do not always accurately reflect in vivo protein distribution. Experimental approaches such as transient expression of fluorescent protein fusions would be necessary to determine the actual subcellular localizations of MsMAG2 proteins. Promoter analysis further suggested that *MsMAG2* genes may respond to multiple developmental and environmental signals. The enrichment of cis-acting elements related to hormone response, light response, and stress response implies that these genes may participate in the integration of endogenous and exogenous regulatory cues. Cis-regulatory elements are important determinants of transcriptional responsiveness and can contribute to expression divergence and functional differentiation among duplicated genes [40,41,42]. This interpretation is broadly consistent with previous reports showing that *MAG2* and *MAL*-related genes are involved in plant growth, hormone responses, and stress adaptation [28,43,44]. However, it is important to note that hormone and stress responses in plants are cell-type-specific and spatially regulated. The presence of cis-regulatory elements in promoter sequences indicates potential transcriptional responsiveness, but does not provide information about the specific cell types, developmental stages, or physiological contexts in which these genes are actually regulated. Additionally, the distinction between endogenous and exogenous hormone signaling should be considered; while promoter analysis identifies elements that may respond to exogenous hormone application, this does not necessarily reflect the gene’s response to endogenous hormonal signals under normal developmental conditions. Therefore, the regulatory roles of *MsMAG2* genes under different physiological conditions require further experimental validation, such as expression profiling under hormone and abiotic stress treatments.

Among the seven identified family members, *MsMAG2L2* was detected by GWAS as a candidate gene associated with tillering traits. This finding is noteworthy because it suggests a possible link between the MAG2 family and branch development in alfalfa. Tillering and branching are complex developmental traits regulated by genetic programs, hormone signaling, and environmental cues [45,46]. Given the established role of MAG2-related proteins in vesicle trafficking and membrane transport, it is reasonable to speculate that MsMAG2L2 may influence tillering through the transport or localization of developmental regulators. In support of this possibility, the predicted protein–protein interaction network based on the Medicago truncatula homolog indicated associations with proteins related to membrane fusion and vesicle transport. Nevertheless, these inferences remain preliminary. The GWAS association identifies *MsMAG2L2* as a candidate gene, but does not demonstrate that this gene controls tillering. Direct evidence from expression analysis, gene editing, or overexpression studies would be required to establish a functional role. In addition, the PPI network presented here is based on computational prediction and provides a hypothesis-generating resource for understanding potential interaction partners of MsMAG2L2. However, as with all in silico interaction predictions, these results require experimental verification to confirm whether the predicted interactions occur in planta. The specific biological function of MsMAG2L2 in alfalfa tillering should be confirmed through additional analyses, including gene expression assays, subcellular localization experiments, and functional verification in transgenic or gene-edited materials.

Taken together, the results indicate that the MAG2 family in alfalfa is evolutionarily conserved but has undergone expansion during genome evolution. The structural features, promoter composition, and GWAS results collectively suggest that *MsMAG2* genes may participate not only in conserved vesicle trafficking-related processes but also in the regulation of agronomically important traits such as tillering. These findings provide a useful framework for further studies on the biological functions of *MAG2* family genes in alfalfa.

## 5. Conclusions

In this study, the *MAG2* gene family was identified in *M. sativa*, and seven members were classified into the MAG2 and MAG2L subfamilies. Phylogenetic and chromosomal analyses showed that these genes are unevenly distributed across the genome, with segmental duplication likely driving family expansion. Conserved motif and gene structure analyses indicated that the MAG2 family is evolutionarily conserved in *M. sativa*, and Motif 10 is a characteristic feature of the MAG2 subfamily. Notably, GWAS analysis identified *MsMAG2L2* as a candidate gene significantly associated with tillering traits, suggesting that it may participate in the regulation of branch development in *M. sativa*. However, it should be noted that the gene analysis in this study was conducted theoretically and requires further experimental confirmation.

## Figures and Tables

**Table 1:** Analysis of basic information about the *MsMAG2* gene family.

ID	Amino Acid Length (aa)	Isoelectric Point	Molecular Weight (Da)	Subcellular Localization Prediction
MsMAG2-1	798	5.56	90,114.58	chloroplast
MsMAG2-2	798	5.52	90,147.62	chloroplast
MsMAG2-3	838	5.89	94,896.24	chloroplast
MsMAG2-4	798	5.56	90,153.59	chloroplast
MsMAG2L1	832	6.66	95,627.39	nucleus
MsMAG2L2	505	5.73	59,022.97	peroxisome
MsMAG2L3	839	6.22	96,895.75	nucleus

## Data Availability

Data are contained within the article.
